# Procedural training for internal medicine residents pursuing subspecialty training: a national survey of fellowship program directors

**DOI:** 10.1080/10872981.2025.2499050

**Published:** 2025-04-30

**Authors:** Effie Singas, Julie Schwartzman-Morris, Matthew J. Whitson, Humza Bashir, Sonia Jacome, Karen A. Friedman

**Affiliations:** a Department of Medicine, North Shore University Hospital-Long Island Jewish Medical Center, Zucker School of Medicine at Hofstra-Northwell Health, New Hyde Park, NY, USA; b University of Toledo Medical School, Toledo, OH, USA; c Department of Community and Population Health, Northwell Health, New Hyde Park, NY, USA

**Keywords:** Internal medicine procedural training, internal medicine subspecialty readiness, ABIM training requirements, graduate medical education, procedures

## Abstract

In 2019, the American Board of Internal Medicine (ABIM) changed procedural requirements for internal medicine (IM) residents, emphasizing that IM residents should ‘have the opportunity to develop competence in procedures which will further their development as fellows in their chosen subspecialty’. While residents need to perform procedures, ‘not all residents need to perform all procedures’. We sought to identify which procedures IM fellowship directors (FD) prefer graduating residents entering their fellowships have experience with and competence to perform. A total of (*N* = 1,463) FDs in the fifteen subspecialties of medicine were identified through the ACGME website and reached via email with a REDCap link to access the survey. The survey was developed amongst the primary authors and included demographic questions and a list of procedures. For each procedure listed, FDs were asked to indicate whether incoming fellows should have knowledge and understanding of, some experience but not competence in, or competence to perform the procedure. The survey also included Likert scale questions aimed at understanding FD attitudes regarding the value of learning procedures during IM training and a free text response soliciting their opinion on the ABIM change in procedure requirements. A total of 424 surveys were completed by FDs from the 15 ABIM subspecialties. Most of the FDs in 8 of 15 subspecialties indicated they preferred incoming fellows have competence in 1–10 (mean 5) of 19 procedures listed and these varied by specialty. One hundred free text responses were received and assigned to one or more themes. This survey can provide guidance to IM program directors and residents applying to subspecialties to tailor their procedural training to the specialty of their choosing.

## Introduction

Training in diagnostic and therapeutic procedures is essential to medical training and patient care. Over the last two decades there has been a significant change in program requirements for procedural training by both the American Board of Internal Medicine (ABIM) and the Accreditation Council of Graduate Medical Education (ACGME). These changes include both the required procedures necessary to complete training and the methodology used to teach procedures. In the recent past, Internal Medicine (IM) residents had a specified number of procedures in which they had to be trained to graduate. Prior to the 2019 academic year, these included drawing venous blood, drawing arterial blood, advanced cardiac life support, pap smear/endocervical culture and placing a peripheral venous line [1]. This bedside procedural training most often was done by senior residents training junior residents, with limited attending supervision, following the ‘see one, do one, teach one’ approach [2]. Competency was thus defined purely by procedure number and results of this approach were varied and not consistent [3]. Furthermore, most residents were uncomfortable performing procedures even when they reported having performed the minimum number required by the ABIM [4,5]. The most recent Common Program Requirements released by the ACGME in July 2021 state that ‘Residents must be able to perform all medical, diagnostic, and surgical procedures considered essential for the area of practice’ [6]. The ABIM now emphasizes that not all residents need to perform all procedures, but that ‘residents must have the opportunity to develop competence in procedures which will further their development as fellows in their chosen subspeciality, or as independent practitioners in their intended fields’ [7]. Therefore, IM residents must now be given the opportunity to develop competence in those procedures most relevant to their future career paths. They must also be able to obtain informed consent, apply standard precautions, establish a sterile field, and apply local anesthetic. Traditionally, many IM residents go on to pursue subspecialty training. In the 2021 fellowship match there were 6368 certified positions [8]. As of 2024, the National Residency Match Program (NRMP) reports that 87.6% of IM graduates pursue subspecialty training. Procedural based fields have the highest number of applicants [9]. IM training program directors (PD) must now tailor their IM procedural training to meet the needs of those residents entering subspecialty fellowships. There is currently no resource available to IM PDs detailing which procedures will benefit residents going into specific fellowship programs.

This survey seeks to identify which procedures IM fellowship directors (FD) prefer graduating residents entering their fellowships have experience with and competence to perform. With this information residency program directors can tailor their trainees’ procedure curriculum to their desired field of interest.

## Methods

A survey was developed amongst the primary authors that included an IM PD, a gastroenterology FD, a pulmonary critical care FD and a rheumatology subspecialty education director. The survey was tested for clarity and face validity by members of the research team. The survey included demographic questions about the FD, the fellowship program and a list of procedures. For each procedure listed, FDs were asked to indicate whether incoming fellows should have only knowledge and understanding of, some experience but not competence in, or competence to perform the procedures. Competency was not defined as it is generally determined by individual programs [10]. Procedures included those that were previously required by the ABIM, commonly performed in the hospital and included the use of ultrasound for diagnosis and guidance of central line placement, thoracentesis, and arthrocentesis. The survey also included Likert scale questions aimed at understanding FDs’ attitudes regarding the value of learning procedures during IM training as well as a free text response soliciting their opinion on the recent ABIM change in procedure requirements. A pre-planned qualitative analysis of the respondents open-ended comments was undertaken using inductive reasoning. Two of the authors (ES, MW) independently reviewed all responses and then identified themes for the responses. Both authors then compared their independently derived themes, and mutually agreed upon themes and their respective definitions to apply to the comments. Each of the comments was then themed independently by the two authors, results were compared, and if authors disagreed on theme assignment, a third author (KF) adjudicated the disagreements.

The survey was IRB approved at Northwell Health (IRB# 21–0048) and administered using the Research Electronic Data Capture (REDCap). The contact information for the participant FDs was identified through the ACGME website. A total of (*N* = 1,463) potential participant FDs in the fifteen subspecialties of medicine were identified and reached at the time of the survey. Participants received an email including detailed information about the purpose of the survey and the REDCAP link to access the survey. The survey remained open for six weeks from 10 February 2021 to 23 May 2021, with a reminder e-mail sent every week after the initial recruitment e-mail. The survey was anonymous.

Summary statistics were used to report geographic data and responses to closed-ended questions. Comparisons were made among the fifteen programs including, when relevant, using chi-squared tests for statistical significance.

## Results

A total of 1463 surveys were sent out to program directors from 15 subspecialties in IM, and 424 responses were received (29% response rate). Most subspecialties had greater than 15 responses to the survey. On average, there were 8 fellows per program, (mean + SD) and FDs reported serving in their role for an average of 7.4 years. FDs from most subspecialties indicated that their fellows perform procedures except for Geriatrics, Hospice/Palliative Care, and Infectious Disease, majority of which indicated that their fellows do not perform procedures (Table 1). Every region of the United States was well represented in the survey responses (Table 2).

**Table 1.** Demographics by subspecialty.

Fellowship Program	# of FDs contacted	# of FD responders	Years as FD Average	Number of fellows per program Average	Do your fellows perform procedures?	
					Yes	No
Allergy and immunology	60	17	5.9	4	17	0
Cardiovascular Disease	196	47	6.4	13.6	47	0
Critical Care Medicine	36	7	6.8	7	7	0
Endocrinology, diabetes and metabolism	126	45	6.1	4.7	43	2
Gastroenterology	160	43	7.4	11.3	43	0
Geriatric Medicine	99	34	5.7	2.9	11	23
Hematology	1	1	20	17	0	1
Hematology and Medical Oncology	122	38	7.4	12.4	37	1
Hospice and Palliative Care Medicine	143	32	3.5	2.7	8	24
Infectious Disease	126	33	6.2	6.4	4	29
Nephrology	118	40	7.9	7.4	40	0
Medical Oncology	5	1	4	9	1	0
Pulmonary Disease	16	4	8.7	5	4	0
Pulmonary and Critical Care Medicine	152	53	7	12.2	53	0
Rheumatology	103	29	8.3	4.6	29	0
**Total**	**1463**	**424**	**7.42**	**8.1**	**344**	**80**

FD = fellowship director.

**Table 2.** Responders by region.

Region	Number of responders
West	59
Southwest	25
Midwest	96
Southeast	97
Northeast	156

The majority (defined as 50% or greater) of FDs in 8 out of the 15 subspecialties surveyed indicated that they preferred incoming fellows have competence in 1–10 (mean 5) of 19 procedures listed (Table 3). Procedures that FDs indicated they preferred incoming fellows to have achieved competence in prior to starting their fellowship training varied by specialty. (Table 3) For example, the majority of Cardiology FDs indicated they preferred their incoming fellows have achieved competence in arterial lines, central venous lines, drawing arterial and venous blood, peripheral venous line placement, and use of vascular ultrasound for line placement. The majority of Pulmonary/Critical Care Medicine FDs favored incoming competence in abdominal paracentesis, arterial lines, central venous lines, drawing arterial and venous blood, nasogastric intubation, peripheral venous line placement, and vascular ultrasound for line placement. Whereas the majority of Gastrointestinal Disease FDs preferred incoming fellow competence in abdominal paracentesis and nasogastric intubation. The majority of Hematology/Oncology FDs preferred competence only in lumbar puncture. Most Nephrology FDs indicated they preferred competence in central venous line placement and vascular ultrasound for line placement. The majority of FDs in 7 specialties including Allergy/Immunology, Endocrine, Geriatrics, Hematology, Hospice/Palliative Care, Infectious Disease, and Rheumatology, did not expect competence for their incoming fellows in any of the procedures listed (Table 3).

**Table 3.** Percentage of FDs by subspecialty preferring incoming fellow competence for procedures listed (bold indicates ≥50%).

	AI	C	CC	E	GI	G	H	H/O	PC	ID	N	O	P	P/CC	R
Abdominal paracentesis	0	6	**71**	2	**70**	9	0	26	22	12	3	0	**75**	**79**	0
Arterial line placement	0	**81**	**71**	2	14	3	0	5	0	3	8	0	**50**	**70**	0
Arthrocentesis	0	0	0	2	0	24	0	3	9	6	0	0	25	6	17
Central venous line placement	0	**89**	**71**	4	16	6	0	13	3	6	**78**	0	**75**	**75**	0
Drawing arterial blood	0	**81**	**57**	4	12	12	0	8	6	6	0	0	**75**	**87**	0
Drawing venous blood	0	**87**	**57**	4	12	12	0	8	6	6	0	0	**75**	**87**	0
Incision and drainage of abscess	0	0	0	4	7	26	0	3	19	27	0	0	25	4	0
IO placement	0	2	**57**	0	5	3	0	0	3	0	0	0	25	25	0
Lumbar puncture	0	4	**57**	2	7	15	0	**61**	3	33	3	**100**	**75**	49	0
Nasogastric intubation	0	15	**57**	2	**86**	21	0	5	25	3	0	0	**75**	**55**	0
Papsmear/endocervical culture	0	0	0	2	5	32	0	3	3	36	0	**100**	25	0	0
Peripheral venous line placement	12	**70**	**57**	9	30	24	0	21	22	9	13	**100**	**50**	**62**	3
Pulmonary artery catheter placement	0	34	0	0	2	0	0	0	0	0	0	0	0	6	0
Thoracentesis	0	9	14	2	7	6	0	24	9	3	3	0	25	36	0
Thoracic ultrasound (pleural effusion localization)	0	13	14	0	2	0	0	8	9	0	5	0	25	36	0
Vascular ultrasound (for line placement)	0	**72**	**71**	7	12	0	0	3	6	3	**70**	0	**50**	**60**	0
Cardiac Ultrasound	0	32	14	0	2	0	0	0	0	0	0	0	0	6	0
Joint Ultrasound	0	0	0	0	2	3	0	3	3	0	0	0	0	0	7
Abdominal Ultrasound	0	0	0	0	9	0	0	3	9	0	8	0	0	9	0

AI = allergy/immunology, C = cardiology, CC = critical care, E = endocrinology, GI = gastroenterology, G = geriatrics, H = hematology, H/O = hematology and medical oncology, PC = hospice and palliative care, ID = infectious disease, N = nephrology, O = medical oncology, *p* = pulmonary, P/CC = pulmonary and critical care, R = rheumatology.

In response to the survey question, ‘Do you have any concerns about the recent change in procedure requirements for internal medicine residents?’, one hundred responses were received. Of the 100 responses, 16 expressed no concerns and 22 answers were not relevant to the question being asked. The remaining 62 responses were assigned to one or more of the identified 18 themes. The two authors disagreed on theme assignment for 2 comments, which were adjudicated by a 3rd author. Major themes surrounding fellowship director concerns identified included the following: Core Competency Skills, Practical Concerns, Fellow Readiness, Culture Shift, Subspeciality Specific Competency, and Fellowship Responsibility. Representative comments for these themes can be seen in Table 4.

**Table 4.** Qualitative responses.

Themes	Number	Percent	Representative quotes
Core Competency Skill	12	11%	Not all residents will practice in a location where radiologists are available 24/7. They need to learn to do simple procedures on their own.
Practical Concern	10	9%	Most of the procedures done in hospital are never done again if you go into primary care or an ambulatory specialty, so you cannot even maintain credentials as you never do central lines or LPs etc after residency.
Fellow Readiness	10	9%	I worry trainees entering into fellowship will not be able to ‘hit the ground running’ in terms of feeling comfortable with placing central lines, arterial lines – things needed to be proficient in the intensive care setting.
Culture Shift	7	6%	Many of our fellows are coming in with little to no experience placing central lines. As a field, Nephrology has largely had to shy away from having fellows placing all central lines; a decade ago, virtually all fellowships had fellows placing scores of lines during their fellowship. This is a crucial area of training which has gone by the wayside due somewhat to time requirements, but mostly due to lack of trainee expertise placing lines prior to fellowship, and I do not see it changing in the near to mid future.
Subspecialty Specific Competency	5	5%	Knowledge and understanding of risks and benefits of procedures are necessary and mandatory. Performing them is best left to people that do them everyday. It is ridiculous to train residents to perform procedures they may never do in their career. Exceptions are general surgery residency and other procedural specialties.
Fellowship Responsibility	5	5%	We typically bring our fellows to a level of competence for appropriate procedures in the first several months of fellowship and will get them where they need to be.

## Discussion

The performance of procedures is an important part of training in IM. In the latest guidelines for training, the ABIM has established a minimal requirement of procedural competency for graduating residents. While ABIM encourages all IM residents to perform procedures, it has tasked programs to provide residents with the opportunity to achieve competence in procedures that will further their development as subspecialty fellows or as independent practitioners in IM.

This is the first survey, to our knowledge, of IM subspecialty FDs’ attitudes towards procedural competency since the ABIM changed their requirements for procedural training. This survey can serve as a guideline for procedural training for IM residency PDs and IM residents pursuing subspecialty training.

As expected, the procedures that FDs preferred varied among the 15 subspecialties. FDs from subspecialties that care for patients in critical care units such as cardiology, critical care medicine, pulmonary and pulmonary/critical care medicine preferred competence in many procedures. Other subspecialty FDs preferred competence in specific procedures relevant to their fields such as paracentesis and nasogastric intubation for gastroenterology FDs, lumbar puncture for hematology/oncology FDs, central venous line placement and vascular ultrasound to guide central line placement for nephrology FDs.

The change in procedural competency requirements has evolved as a result of concerns for patient safety and work hour limitations leading to time constraints [11]. As procedural competency requirements have changed, IM residents have become less comfortable performing common bedside procedures [12]. While some FDs expressed the opinion that procedures were best left to experts and that most IM residents would not be performing procedures in their practice, most FDs in this survey indicated that performing procedures was safe and that patient care, patient ownership and ability to obtain informed consent would improve.

There are several limitations to this study. Our small response rate is not necessarily representative of the opinions of the entire group of subspecialty fellowship directors. However, even with a lower response rate there was a good distribution across specialties and geographic areas allowing the results to be generalizable. Our survey at this time was limited to subspecialty fellowship directors, but we recognize that residents entering primary care and hospital medicine are expected to have procedural competence as well. We were not able to link individual responses to regions of the country, and therefore could not detect regional differences among the different specialties. In more remote areas of the country where subspecialty programs are fewer in number, internal medicine residents may be learning many of these common procedures prior to starting their fellowship training. We also did not ask fellowship directors if residents competence in performing procedures impacted their fellowship candidate selection. As procedural training is so variable amongst programs, the authors which included fellowship directors do not believe that procedural competence has a role in fellowship candidate selection. Finally, in terms of our qualitative analysis, the largest category of comments was uninterpretable and difficult to assign to one of the identified themes. There were several program directors that were unaware of the recent ABIM change to procedural requirements.

## Conclusion

The recent change in procedural requirements by the ABIM mirrors the change in practice over the last two decades by internal medicine residents and practicing internists. These changes have resulted from patient safety concerns and work hour limitations. While the ABIM has clearly stated that residents should have the opportunity to develop competence in procedures that will further their development as independent practitioners in their chosen fields of practice after residency, there is no longer a specific requirement to learn core procedures as there had been in the past. It is now up to the individual program directors to tailor procedural training for their residents. This survey can provide some guidance to internal medicine program directors and residents applying to subspecialties to tailor their procedural training to the specialty of their choosing. Further surveys should aim to identify those procedures which will benefit trainees entering primary care and hospital medicine.
